# Brain structural evidence for a frontal pole specialization in glossolalia

**DOI:** 10.1016/j.ibror.2020.06.002

**Published:** 2020-06-23

**Authors:** Yoshija Walter, Sebastian Dieguez, Michael Mouthon, Lucas Spierer

**Affiliations:** Neurology Unit, Medicine Section, Faculty of Science and Medicine, University of Fribourg, Fribourg, Switzerland

**Keywords:** Glossolalia, Expertise, Language control, Brain structure

## Abstract

Glossolalia is defined as the ritual oral production of phoneme sequences without recognizable semantic content. The functional underpinnings of glossolalia, and notably whether it consists of a highly specific or ordinary behavior, remain largely unresolved. We addressed this question by measuring the structural brain remodeling associated with the extensive practice of glossolalia in thirty experts. This approach enabled us to circumvent the limitations of functional imaging to reveal the neural correlates of behaviors elicited in specific contexts and involving movements incompatible with most imaging methods.

Whole-brain regression analyses of glossolalia expertise with indices of grey and white matter structure revealed positive associations between practice time and grey matter volume within the left frontal pole and the right middle frontal gyrus.

These findings suggest that glossolalia involves a degree of neurocognitive specialization, though not at the level of language control and production networks, but within domain-general executive areas. They further call for including multi-tasking and interference suppression as key processes in models of unrecognizable speech production. Our results also concur with current demonstrations that measures of brain structural remodeling may help identifying whether cognitive skills depend on networks specialization or on a recycling of already existing processes.

## Introduction

Glossolalia is defined as the ritual oral production in a religious context of phoneme sequences without recognizable semantic content. The functional underpinnings of glossolalia remain largely unclear due to the difficulty in triggering these processes in laboratory setting, and because the articulatory movements it involves create motion artifacts incompatible with most functional neuroimaging methods. Yet, understanding the functional organization of glossolalia would help solving the longstanding debate between normative and descriptive approaches in religious studies, which respectively tended to pathologize glossolalia as a behavior linked to trance, schizoid and epileptic states (Goodman, 2008) or to approach it in more functional terms as a socially learned behavior involving no specific skill or dysfunction (Pozzo, 2013).

Here, we addressed these questions by examining the brain anatomic remodeling associated with extensive practice of glossolalia: While evidence for targeted neuroplasticity in the acquisition of glossolalia would not, per se, suggest that the behavior is pathological or irrational, it would rule out the possibility that glossolalia is a perfectly ordinary behavior which does not require any acquired functional specialization. In addition, the location of any plastic modification associated with glossolalia practice would provide important theoretical insights into the nature of this controversial behavior. Motor and cognitive expertise have indeed been found to be associated with specific neuroplastic changes, such that the acquisition of certain behaviors and skills may not be accessible in the absence of those structural modifications (e.g. Chang, 2014). As a notable example, structural modulations have already successfully revealed the necessity to acquire specialized neurocognitive processes in meditation, a practice relevant to glossolalia for its spiritual content and the involvement of executive control of ongoing cognitive processes. A recent review by Fox et al. (2014) indeed reports small to large positive associations between anatomic remodeling and meditation expertise (Cohens’ d of 0.3–1.7 for modulations in grey matter volume with mediation; Fox et al., 2014). If this is also the case with glossolalia, it would then be akin to specialized processes unavailable to novices and only underlying especially skilled individuals. We call this the *specificity hypothesis*: glossolalia would be a learned behavior that induces neurocognitive remodeling (Draganski and May, 2008).

On the other hand, if glossolalia does not involve specific neural reorganization through extensive practice, it would confirm a *mundanity hypothesis*: glossolalia merely requires “at hand” neurocognitive functions, already available for other purposes, in particular language control processes. This latter hypothesis would be in line with linguistic evidence that the structure of glossolalia closely mimics the structural patterns of the glossolalics’ ordinary tongue. On this view, “speaking in tongues” would in fact mean producing meaningless utterances using regular language and executive functions, but would appear more dramatic than it is because the verbal behavior is embedded in a sophisticated religious context. Note that these hypotheses are offered regardless of the practitioners’ belief that glossolalia is a supernatural feat “given” by the Holy Spirit, which is irrelevant to our purposes.

To identify the brain regions involved in glossolalia and the degree of specificity of this practice, we recruited a group of 30 individuals from a homogenous community of Christians from Pentecostal churches with extensive glossolalia practice, and recorded the affective and control subjective experience they associate with this practice, as well as their general religiosity. We further recorded brain structural and diffusion tensor imaging, and applied a whole-brain regression of glossolalia expertise (as indexed by the total practice time) on grey matter volume and thickness, as well as white matter fractional anisotropy. While we could generate predictions on the most likely loci for practice-induced plastic remodeling based on current neurocognitive models of language production and control, we felt that a first-pass exploratory whole-brain investigation was more appropriate given the current lack of systematic neural investigations of glossolalia. We thus do not limit our investigation to a limited number of loci of interest, although we expect to find associations in the network described below.

The production of random phonemes without any semantico-syntactic organization requires an inhibitory control of the prepotent tendency for producing real-language phonemics and syntactic configurations. According to the *specificity* hypothesis, the repeated practice of glossolalia may thus have induced plastic modifications within the left fronto-temporo-parietal network controlling language production. This hypothesis more precisely predicts structural modifications within the anterior cingular executive areas controlling language production and within the dorsal phonological production stream, notably including the left inferior frontal gyrus, temporo-parietal junction and the superior longitudinal fasciculus (Abutalebi, 2008). The arcuate fasciculus may also be involved since it connects the caudal part of the superior temporal gyrus to the frontal lobe (Nieuwenhuys et al., 1988), which supports bidirectional functional interactions between left inferior frontal spoken word production and temporo-parietal phonemic retrieval and sequencing areas (Matsumoto et al., 2004). Finally, beyond the control networks also typically involved in bilinguals, glossolalia may require militasking and inhibiting usual language production. Corresponding mechanisms are for instance at play in simultaneous interpreters and are thought to depend on the frontal poles in addition to ventrolateral prefrontal areas (Becker et al., 2016).

## Methods

### Participants

Thirty healthy experts in glossolalia (i.e. with a frequent and longstanding practice of glossolalia) participated in this study. The group included 13 males and 17 females aged 29.6 ± 4.6 years (mean ± SD), and four left-handed individuals (Oldfield, 1971). The participants were all confessing Christians from Pentecostal churches of the same country. The practice of glossolalia is a regular part of their Charismatic-Christian faith and the teaching as well as the modes of practice were comparable in all the participants. The glossolalic productions were of course different and idiosyncratic across participants, but they were still homogeneous at the level of their main properties (phonemes, length, etc.), although we have no detailed data on these linguistic aspects. None of them modified their practice of glossolalia for the purpose of our study: all practiced the spiritual ritual for a different amount of time though with a corresponding intensity. Interviews and self-rankings were used to select and assess the nature of their glossolalic practice, and the religiosity questionnaire was selected from the validated approach by Huber and Huber (2012).

**Fig. 1:**
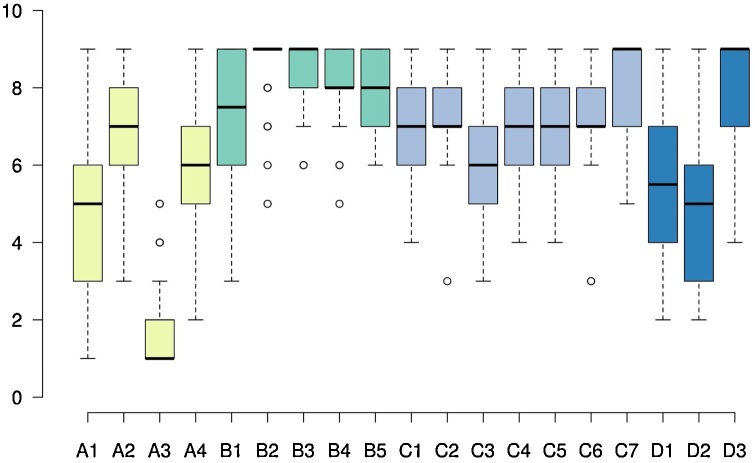
Glossolalic practice assessment; “Prayer” is used in the questionnaire to refer to the individual practicing glossolalia since our study focuses on this practice in a religious context. The box plot for each assessed dimension as. A. Control. A1. Feeling in control over the practice (1: I’m in full control / 9: I’m completely seized by the Holy Spirit); A2. Who formulates the words? (1: I formulate them myself / 9: The Holy Spirit formulates every word through me); A3. Deciding on the start and stop point of the prayer. (1: I can control this / 9: It’s out of my control). A4. Controlling which words ‘flow out’. (1: I can control the words / 9: I cannot control the words). B. Difficulty. B1. Difficulty to switch from mother language to another language. (1: Very hard / 9: Very easy); B2. Difficulty to switch from mother language to glossolalia. (1: Very hard / 9: Very easy); B3. Is it easy for you to pray in tongues? (1: Very hard / 9: Very easy); B4. Fluency of glossolalia. (1: I’m very stagnant / 9: I’m very fluent); B5. Confidence in practice. (1: Very unconfident / 9: Very confident). C. Experience. C1. Importance of glossolalia for the faith. (1: Not at all important / 9: very important); C2. Feeling closer to God after glossolalia. (1: never / 9: almost always); C3. Hearing God during glossolalia (1: never / 9: almost always); C4. Feeling God’s presence during glossolalia. (1: never / 9: almost always); C5. Felt intensity of glossolalic prayer. (1: not at all intense / 9: very intense); C6. Felt intimacy of glossolalic prayer. (1: not at all intimate / 9: very intimate); C7. Comfortableness while praying in tongues. (1: Very uncomfortable / 9: Very comfortable). D. Linguistics. D1. Is your glossolalic prayer simple or complex? (1: very repetitive / 9: very diverse); D2. Does your glossolalia sound like a real language? (1: Does not sound like a normal language / 9: Sounds like a normal one); D3. Resemblance of glossolalia to mother language. (1: Sounds like my mother language / 9: Sounds like a foreign language).

The group total practice time was estimated based on participants’ self-reports: they had practiced an average of 230 h, SD ±275 h (3−1299 h range), over 11.9 year ± 4.8 years (0.75−20y range), with a frequency of practice of 11.0 ± 8.9 times per week, for 2.1 ± 1.8 min per session. Questionnaire surveys confirmed that our participants were highly religious (4.6 ± 0.3 (3.7; 5.0) Mean religiosity ± SD (min; max), Max = 5; Centrality of Religiosity Scale CRS-15 (cf. Huber and Huber, 2012, Table 1 for details) and that the practice of glossolalia was automatic and effortless (Fig. 1).

**Table 1:** Centrality of Religiosity questionnaire.

Centrality of Religiosity Scale CRS-15	Mean ± SD and range (min; max) Max = 5
Mean religiosity of participants	4.6 ± 0.3 (3.7; 5.0)
Intellect	4.3 ± 0.2 (3.3; 5.0)
Ideology	5.0 ± 0.0 (5.0; 5.0)
Public Practice	4.6 ± 0.2 (2.3; 5.0)
Private Practice	4.7 ± 0.1 (3.7; 5.0)
Experience	4.0 ± 0.1 (3.0; 5.0)

The centrality of religiosity for each participant was calculated using three variables per dimension (abiding by the standardized and validated model of Huber and Huber, 2012). The scale ranges from 1= “not religious” to 5= “highly religious”. ‘Intellect’ assesses how much a person thinks about religious questions;’ ideology’ : the presence of religious opinions and beliefs;’ public practice’: how strongly the faith is practiced in communion with others;’ private practice’: the integration of a personal prayer life;’ experience’ : how strongly one feels to be in contact with the divine.

We further assessed with a custom-made questionnaire four main aspects of the glossolalia practice: Whether the individuals felt in control during glossolalia, whether it was difficult, how the experience was and what they thought about the output of the practice. The group-averaged answers are reported in Fig. 1.

### Data acquisition, preprocessing and analyses

The aim of the MRI (grey matter volume and cortical thickness) and DTI (fractional Anisotropy) whole-brain analyses was to investigate the association between brain structure and the glossolalia expertise (total practice time) by regressing the total practice time on the anatomic dependent variables. We chose a regression approach and not a comparison between the glossolalia experts and a control group because the variability in expertise in the glossolalia group we had access to was large and their socio-cultural homogeneity allowed for a good control of the factors potentially confounding our effect of interest.

### MRI data acquisition

MRI data was acquired with a 3 T MRI scanner (Discovery MR750; GE Healthcare, Waukesha, WI) equipped with a 32-channel standard head coil. We recorded and analyzed grey matter structure with Voxel-Based Morphometry and Surface-Based Morphometry using well-established procedures and toolboxes of SPM 12 (Statistical Parametric Mapping, Institute of Neurology, London, UK) running on MATLAB R2016b (MathWorks, Natick, MA, USA). White-matter structure was examined using the Tract-based spatial statistics (TBSS) approach on the DTI data (Smith et al., 2007) implemented in the FSL 5.0.10 software (FMRIB software library; Jenkinson et al., 2012).

The head of the participants was maintained by a sound-attenuating memory foam to reduce movements. A T1-weighted image (FSPGR BRAVO sequence) was acquired to examine grey matter anatomy, using the following parameters: 280 coronal slices, voxel size: 0.86 × 0.69 × 1 mm, matrix size: 256 × 256, FOV = 22 × 17.6 cm, TR =7300 ms, TE =2.8 ms, prep time=900 ms, flip angle = 9°, parallel imaging acceleration factor (PIAF) = 1.5, intensity correction: PURE. In addition, a Diffusion Tensor Imaging (DTI) sequence was acquired to study white matter tractography with the following parameters: 60 axial slices, acquisition interleaved voxel size: 2 × 2 × 2 mm, matrix size: 128 × 128, FOV = 26 × 26 cm, inter-slice spacing = 0.2 mm, TR =8000 ms, TE =87 ms, flip angle = 90°, PIAF = 2, 60 non-collinear directions with b-value = 1000s/mm2, five b = 0 images. In order to correct the distortion of the static magnetic field during post-processing of the DTI, five supplementary b = 0 images were acquired with an opposite phase encoding direction.

### Grey matter analysis

The grey matter structure was characterized at the level of its volume via Voxel-Based Morphometry (VBM), and of the cortical thickness via Surface-Based Morphometry (SBM). Both analyses share common preprocessing steps using automated procedures in Computational Anatomy Toolbox (CAT12.5; the Structural Brain Mapping group, Jena University Hospital, Jena, Germany) implemented in SPM12 (Statistical Parametric Mapping, Institute of Neurology, London, UK) running on MATLAB R2016b (MathWorks, Natick, MA, USA). First, the T1-weighted images were visually inspected, and origin set on the anterior commissure. After bias correction, T1 image were segmented into gray matter (GM), white matter (WM) and cerebrospinal fluid (CSF; Ashburner and Friston, 2005). After this step, additional visual and sample homogeneity checks were performed.

For the VBM analysis, GM probability maps were modulated to preserve relative volumes after spatial registration to MNI space based on the build-in template provided in the toolbox (Ashburner, 2007; Kurth et al., 2015). The resulting images were smoothed with an 8 mm FWHM isotropic Gaussian kernel. These data were then analyzed with a multiple regression random effect approach (RFX) with the age, sex, Edinburgh index of handedness, total intracranial volume (TIV, computed with CAT12 tool) as confounding factors, and the glossolalia expertise index (total practice time) as the variable of interest.

For the SBM analysis, the tissue segmentation was used in the CAT12 toolbox to estimate the distance between the inner surface (WM/GM interface) and the outer surface (GM/CSF), a distance corresponding to the cortical thickness. The local maxima of this distance was then projected onto other neighboring GM to create a cortical thickness map (Dahnke et al., 2013). This approach allows handling partial volume information, sulcal blurring, and sulcal asymmetries without explicit sulcus reconstruction. For inter-subject comparisons, cortical thickness maps were resampled into a common coordinate system and smoothed using a 15 mm Gaussian heat kernel (Yotter et al., 2011; Zhuang et al., 2017).

The empirical quality rating of the raw T1 images based on the resolution, noise and bias was assessed by the CAT12 toolbox as a weighted average index in the 83–88 % range (mean ± SD = 86.1 ± 0.94 %) indicating good quality data. A whole brain statistical analysis on these data was performed with a multiple regression RFX with the age, sex, Edinburgh index of handiness as confounding factors, and the glossolalia expertise index as the variable of interest.

All the voxelwise analyses used a statistical threshold of p < 0.05 FWE corrected for multiple comparison after non-parametric estimation with the Threshold-Free Cluster Enhancement (TFCE) toolbox (v174, the Structural Brain Mapping group, Jena University Hospital, Jena, Germany). Results were localized with the Neuromorphometrics atlas of SPM12.

### White matter analysis

The WM was examined using the Tract-based spatial statistics (TBSS) approach on the DTI data (Smith et al., 2007) implemented in the FSL 5.0.10 software (FMRIB software library, Jenkinson et al., 2012). To correct the distortion in the diffusion-weighted images, b0 images were collected with reversed phase-encode blips, resulting in pairs of images with distortions going in opposite directions. From these pairs, the susceptibility-induced off-resonance field was estimated by combining these b0 values using the TOPUP tool implemented in FSL (Andersson et al., 2003). The EDDY tool then used it as input to unwarp the DTI images. In addition, they were affine-aligned to the mean of b0 images and corrected for eddy-current artefacts. Then, after the generation of a binary brain mask based on the reference b0 image using the BET tool with a 0.2 threshold, the diffusion tensor was fitted to the data to compute the fractional anisotropy (FA) diffusion index with the DTIFIT tool of the FDT toolbox. All processed FA data were sent to a TBSS pipeline (Smith et al., 2007): nonlinearly transformed on the mean FA template (FMRIB58_FA) and then affine transformed on the standard MNI space. The resulting images were used to create the study-specific mean FA image, which was skeletonized with a threshold FA > 0.2 to generate the common white-matter tract skeleton map. Finally, individual FA images were projected onto this reference skeleton.

We assessed whether the anatomic WM connectivity in the whole brain correlated with the expertise index by conducting a multiple regression RFX analysis with the age, sex, Edinburgh index of handiness as confounding factor, and the glossolalia Expertise index as the variable of interest. Statistical inference of the voxelwise analysis was based on the permuted p-values (5000 permutations; Nichols and Holmes, 2002), which included the threshold-free cluster enhancement (TFCE) with a threshold of p < 0.05.

Results

### Grey and white matter structure

**Fig. 2:**
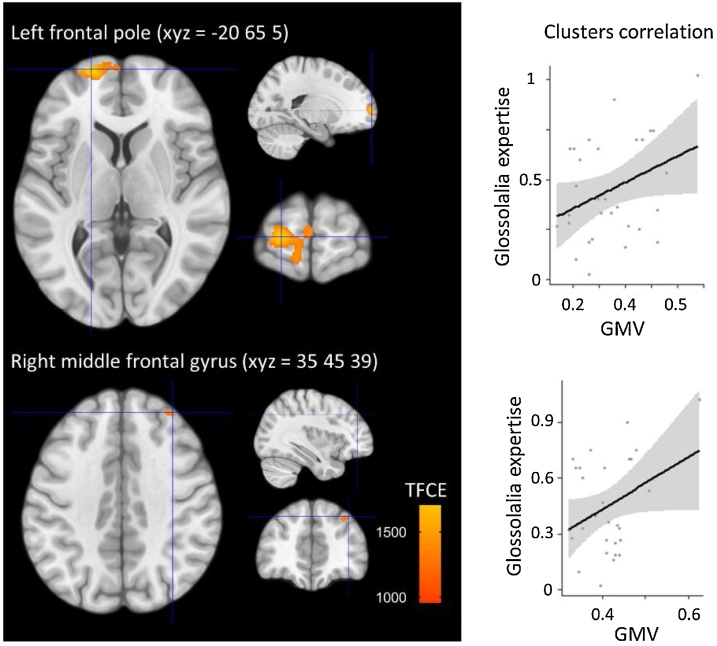
Whole brain analysis of grey matter volume with voxel-based morphometry. The left panel displays two brain clusters showing significant correlations with the glossolalia expertise index. The contrast represents the threshold-free cluster enhancement (TFCE) statistic at p_FWE_<0.05 combined peak-cluster corrected for multiple comparison threshold. The right panel displays the correlation scatterplots for these clusters. GMV = grey matter volume index.

The whole-brain voxelwise VBM analysis revealed a positive correlation between grey matter volume and glossolalia expertise in the left frontal pole region and the superior frontal gyrus (MNI xyz = -20 65 5, p_FWE_<0.05 combined peak-cluster corrected for multiple comparison estimated with TFCE, directional, size = 746 voxels) as well as a second cluster centered on the right middle frontal gyrus (MNI xyz = 35 45 39, p_FWE_<0.05, size = 40 voxels; Fig. 2).

Analyses of cortical thickness did not reveal any association between glossolalia expertise and cortical thickness (p_FWE_>0.05 combined peak-cluster corrected for multiple comparison and estimated with TFCE, non-directional).

Likewise, we found no association between glossolalia expertise and white matter tracts fractional anisotropy (p > 0.05 corrected for multiple comparison and TFCE estimated for voxelwise analysis).

## Discussion

We identified a positive association between glossolalia expertise and grey matter volume in the left frontal pole and right middle frontal areas. The finding for at least one locus of association between brain structure and glossolalia expertise speaks against our ‘mundanity hypothesis’ and suggests that glossolalia requires some degree of brain specialization. More precisely, the result for a remodeling of the frontal poles suggests that glossolalia involves high-level executive control of goal-oriented behavior, such as multitasking (Gilbert et al., 2006) and switching between attending to internal vs external information (Burgess et al., 2007). The role of this region in supporting expertise in language-related switching is further supported by the finding of Becker et al. (2016) of an increase in left frontal pole grey matter density in simultaneous interpreters.

Also consistent with the hypothesis of an involvement of executive control to prevent ‘real’ words to interfere with the production of glossolalic speech, the right middle frontal gyrus has been involved in interference suppression and response inhibition (Garavan et al., 1999). This finding further suggests a potential role for executive control of speech during glossolalia, and points toward a need for suppressing interference from usual language and switching rather than for generating new speech patterns in this practice.

Our results for prefrontal remodeling with glossolalia expertise may also indicate the involvement of any type of behavior less directly involved in glossolalic speech and more to the ritual practice per se, such as feeling detached from one’s direct surroundings in order to focus on what is perceived and interpreted as a supernatural gift, or living on a daily basis with potentially costly social practices and behaviors, that are poorly understood by the general public. In line with this assumption, our results further revealed an absence of specialization within language control and production areas, which may appear surprising given the key involvement of this system in producing unrecognizable phoneme sequences. We investigated this null result by regressing the value of the Glossolalic practice assessment item on the linguistics dimension of prayer (D1), which did not show any association with grey matter structure. This follow-up analysis support the idea that glossolalia loads more on executive than linguistic components.

In any case, our findings speak against the widespread idea that glossolalia is effortless and uncontrolled (Pozzo, 2013; Chouiter and Annoni, 2018), although recurrent practice may well have partly automatized the executive processes involved. Our questionnaire results indeed point to some ambiguity on the issue, with participants rating an average midpoint on whether they or the Holy Spirit are in control of their utterances and whether they can select or not the “words” they utter, while claiming full control on the initiation and ending of glossolalia (Fig. 1).

As a main limitation of the study, we could not rule out that other factors also correlating with the total glossolalia practice time may have mediated our results. For instance, glossolalia expertise is likely associated with the amount of participants’ Christian/ church activity. Yet, given that such activity involves a high diversity of neurocognitive processes, this factor would unlikely have resulted in focal structural changes as those we observed; even if glossolalia may indeed be supported by variable idiosyncratic processes in our group, it still involves a limited number of language control and production processes as compared to those related to church activities. Second, we would note that while we had specific predictions on the neurocognitive processes involved in glossolalia and thus potentially susceptible to plastic changes with practice, our interpretation of the results largely depends on reverse inference. While such interpretations have important shortcomings (Poldrack, 2006), we feel that in the present study it still helpful in establishing an initial model of glossolalia by pointing out important neurocognitive aspects supporting this underexplored practice. As such, while we provide robust, conservatively corrected results, our study should be considered as a first-pass, semi-exploratory investigation of the neural underpinnings of glossolalia designed to open future confirmatory works.

We would further note that we did not find any association between glossolalia expertise and white matter fractional anisotropy. While we cannot rule out that this null result followed from a lack of statistical power, it suggests that anatomic connectivity is not critical for glossolalia. Importantly, similar patterns of grey matter changes without modification in white matter have been reported in the close field of structural modifications in bilingualism (e.g. Mechelli et al., 2004).

We tentatively conclude that, at least in our population sample, glossolalia does not depend on any specifically acquired language skill, but still requires the development of specialized executive control processes. This pattern overall supports the ‘specificity hypothesis’, while considering that the specialization with expertise took place within a region with a low-specificity domain-general function. With these results in mind, we suggest that although requiring a specific form of control, glossolalia may constitute a case of cultural recycling, for religious and socio-cultural purposes, of neurocognitive speech production processes initially developed for other functions (Anderson, 2014). The absence of any detectable modulation of such areas through extensive practice suggests that glossolalia emerged, and remained stable across centuries and continents, because of an optimal balance between, on the one hand, its dramatic effects on the practitioner and her audience, and on the other hand, its relative ease of acquisition and execution.

## Conflict of interest

The authors declare no conflict of interest.

## Authors contribution

Conception and study design (YW), data acquisition and statistical analysis (YW, MM), interpretation of results (YW, SD, LS), drafting the manuscript work or revising it critically for important intellectual content; approval of final version to be published; agreement to be accountable for the integrity and accuracy of all aspects of the work (All authors).

## Ethical standards

All procedures were approved by our local ethics committee. Each participant provided written, informed consent to participate in the study.

## Funding sources

This work was supported by a grant from the 10.13039/501100001711Swiss National Science Foundation (Grant # 320030_175469 to LS).
